# Open Science Framework (OSF)

**DOI:** 10.5195/jmla.2017.88

**Published:** 2017-04

**Authors:** Erin D. Foster, Ariel Deardorff

## GENERAL DESCRIPTION

The Open Science Framework (OSF) is a tool that promotes open, centralized workflows by enabling capture of different aspects and products of the research lifecycle, including developing a research idea, designing a study, storing and analyzing collected data, and writing and publishing reports or papers. It is developed and maintained by the Center for Open Science (COS), a nonprofit organization founded in 2013 that conducts research into scientific practice, builds and supports scientific research communities, and develops research tools and infrastructure to enable managing and archiving research [1]. As an organization, the COS encourages openness, integrity, and reproducibility in research across scientific disciplines [2]. The OSF supports a variety of tools and services to assist in the research process. This review focuses primarily on the core functionality of the OSF, with brief descriptions of some of the other existing tools and services.

## FEATURES

The core functionality of the OSF is its ability to create and develop projects. Very simply, a project functions as a workspace, with the design of a particular project depending on users and the type of research workflow that they are trying to manage and preserve. Users might wish to set up a project for a particular paper or specific experiment or for the work of an entire lab. To create a project, users must set up a free account with the OSF. Once logged in, users are taken to a dashboard with the option to create a project. The standard project layout includes a wiki, a log of recent activity, and spaces to upload files, add tags, and create new components (i.e., subprojects). Each user, project, component, and file is given a unique, persistent uniform resource locator (URL) to enable sharing and promote attribution. Projects can also be assigned digital object identifiers (DOIs) and archival resource keys (ARKs) if they are made publicly available. The OSF provides built-in version control that records changes to project files and previous versions through OSF Storage.

The OSF is intended to be collaborative, and users can easily add contributors to projects. The OSF supports controlled access, so project members can be assigned different permissions: read only, read and write, and administrator. Contributors do not have to set up an OSF account prior to being added to projects. Unregistered contributors can be added to projects using their full names and email addresses; they will be contacted with a link to set up an OSF account. Contributors who already have an OSF account can be added to a project by searching for their names in the OSF.

While the spirit of open science encourages making projects publicly available, there are options to make all or parts of a project private. The Project Overview page includes a toggle button that allows those with administrator-level permissions on the project to determine which parts of the project (if not all) will be public or private. In general, private projects are not browsable. Users can find public projects online. Certain components of a public project can be made private; those will be hidden from public view. To capture impact, the OSF also includes project-level analytics, such as unique visitors, downloads per project file, and top referrers.

In addition to using unique, persistent URLs, DOIs, and ARKs, the OSF promotes sharing in a variety of additional ways. A primary one is the option to add a license. The COS links to resources for choosing a license with a variety of license options available, including Creative Commons, MIT, Apache, and GNU General Public. A user who does not wish to use any of the predetermined licenses can upload an alternative license. A license can apply to the project as a whole, or different licenses can be assigned to different parts of the project.

While there are many features built into the OSF, the platform also allows third-party add-ons or integrations that strengthen the functionality and collaborative nature of the OSF. These add-ons fall into two categories: citation management integrations and storage integrations. Mendeley and Zotero can be integrated to support citation management, while Amazon S3, Box, Dataverse, Dropbox, figshare, GitHub, and oneCloud can be integrated to support storage. The OSF provides unlimited storage for projects, but individual files are limited to 5 gigabytes (GB) each. Using one of the storage add-ons eliminates this restriction.

Registration is a major feature of the OSF and its efforts to preserve, provide access to, and promote transparency in research. Any OSF project can be registered, which means that a time-stamped version of the project is created that cannot be edited or deleted and is intended to act as a preserved version of a project. A user can, however, withdraw a project, which removes the content of the registered project but leaves behind a record of it. Registered projects can be made public immediately or embargoed for up to four years. Additionally, DOIs and ARKs can be created for public registrations. Any content stored on third-party servers is copied as part of the registration process and stored with the rest of the project content on OSF servers.

## USER COMMUNITY AND SERVICES

The COS supports a diverse audience, from researchers and scientists to software developers to publishers and societies. While many of the features of the OSF are designed to help researchers create, manage, and preserve research, there are additional free OSF tools and services that can engage other user groups.

OSF for Institutions allows institutions to create a landing page in the OSF to identify and connect affiliated users and projects. OSF users can identify their projects as being affiliated with particular institutions, and additional features, such as single-sign on and institutional branding of the landing page, can provide a seamless user experience for institutional affiliates using the OSF.

OSF for Meetings provides a space to share posters and presentations from meetings and conferences. When a user registers a conference or meeting, the OSF will provide a branded, persistent page where conference attendees can upload posters and presentations, as well as browse content added by themselves or colleagues before, during, or after the event.

OSF Preprints lets OSF users share preprints for feedback and to gain exposure. Each preprint receives a unique identifier, and users can upload supplementary files as needed. Users also have access to analytics for their uploaded preprints.

In addition to engaging with user communities through these services, the COS maintains an Ambassador program, which works at local levels to promote and support open science at research institutions. These ambassadors represent the COS and can provide training and additional resources about the OSF or other COS products. A list of current COS ambassadors is available online.

## DOCUMENTATION AND TECHNICAL REQUIREMENTS

As an open source service, the OSF has freely available documentation and support. The OSF home page has a link to support, including FAQs, contact information, and a set of OSF Guides that give step-by-step guidance and instructions on using the OSF. The COS also delivers regular workshops, webinars, and online tutorials. Past webinars can be viewed on the COS YouTube channel.

Access to the OSF requires only a working computer and Internet connection. Larger collaborations such as the OSF for Institutions program require additional configuration by the COS and institutional information technology staff.

## CASE STUDIES

One of the most prominent uses of the OSF tool is the Psychology Reproducibility Study, a collaboration between the University of Virginia and the COS. The more than 270 researchers involved in this project replicated 100 top psychology studies to see if they could produce the same results [3]. While the study results are interesting (they were able to replicate fewer than half), what is more interesting in this context is that the entire research process for each study—including data, analysis, publications, and comments—was openly shared on the OSF [4]. The Psychology Reproducibility Study highlights the strengths of the OSF, including collaboration features, the ability to create subprojects, and citation features, which allow researchers (and authors like us) to cite various components of the project. While this project is probably larger than most using the OSF, it serves as a good example of how health sciences researchers might integrate the tool into their research workflows.

**Figure 1:**
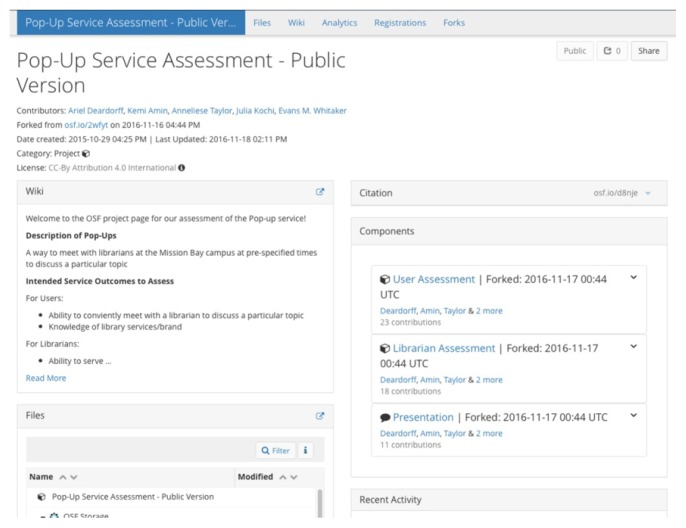
University of California–San Francisco (UCSF) Open Science Framework (OSF) project page Available at https://osf.io/d8nje/.

A smaller-scale example of OSF usage comes from the University of California–San Francisco (UCSF) Library, where, in the fall of 2015, a team of librarians used the OSF for an assessment project [5]. They selected the OSF because they needed a tool that would allow them to easily keep notes, upload files, collaborate, and share information (Figure 1).

While initial response to the OSF was positive, some team members found it confusing to navigate the various components and had trouble locating particular documents or wiki pages. The wiki feature also proved not to be as user-friendly as they had hoped, because the formatting options were limited and editing took some getting used to. Some of these challenges might have been due to the design of the project in the OSF; a more well-thought-out project with less hierarchy might have been easier to navigate. In the end, the team decided that while they liked some aspects of the tool, particularly the ability to assign a DOI to a project, their institutional wiki or Box account would have been a better tool for this project. They did note, however, that the OSF would provide an excellent way to collaborate with colleagues at other institutions who do not have access to UCSF-only tools.

## SUMMARY

Because of its focus on openness and unique identifiers, the OSF can be an excellent tool for promoting best practices around reproducibility, transparency, and research data management. The high degree of flexibility means that projects can be customized easily to fit a variety of needs, from small projects to large research collaborations. Moreover, the COS is continually working to add more components and capabilities to the tool. As with all research tools, the usefulness of the OSF depends on how easily it can be adapted into a researcher’s workflow. The librarians at UCSF found it to be less useful than other tools they had available to them, but the example of the Psychology Reproducibility Study shows how the unique registration and collaboration features can provide a real benefit.

Beyond researchers’ workflows, local institutional requirements or policies can also affect how the OSF can be used. For example, whether or not the OSF is Health Insurance Portability and Accountability Act (HIPAA)–compliant depends upon an institution’s security and privacy practices and would require further conversations with an institution’s information technology administration. Anyone interested in using the OSF is encouraged to create a free account and give it a try. Librarians might also consider inviting a local COS ambassador to give a presentation or contact the COS for a presentation on the OSF as a service or tool.
